# Parental report of language, attention and executive functions at two years: correlational structure of measures and applications to prematurity

**DOI:** 10.12688/wellcomeopenres.24065.2

**Published:** 2026-03-13

**Authors:** Rebekah Smikle, Kadi Vaher, Lorena Jiménez-Sánchez, Amy Corrigan, Helen Turner, Sue Fletcher-Watson, Simon R Cox, Hilary Richardson, James P Boardman

**Affiliations:** 1 Centre for Reproductive Health, Institute for Regeneration and Repair, The University of Edinburgh, Edinburgh, Scotland, UK; 2 Centre for Clinical Brain Sciences, The University of Edinburgh, Edinburgh, UK; 3 School of Philosophy, Psychology, and Language Sciences, The University of Edinburgh, Edinburgh, UK; 4 Salvesen Mindroom Research Centre, The University of Edinburgh, Edinburgh, UK; 5 Lothian Birth Cohorts, Department of Psychology, The University of Edinburgh, Edinburgh, UK

**Keywords:** prematurity, rating scales, language, attention, executive functions, cognition

## Abstract

**Background:**

Parent-report measures are increasingly used in research and clinical settings to assess early cognitive outcomes after preterm birth. Directly observed cognitive measures often share a correlational structure, from which a general factor of cognition is calculable. We investigated associations between gestational age at birth and language, attention and executive function at two years using parent-report measures and examined whether a general factor could be derived from parental assessment of abilities across these domains.

**Methods:**

183 2-year-old children (96 preterm and 87 term) were assessed for language using the Vineland Adaptive Behaviour Scales-3rd edition and the MacArthur-Bates Communicative Development Inventory, attention using the Early Childhood Behaviour Questionnaire, and executive functions using the Behaviour Rating Inventory of Executive Function-Preschool version. Linear regression was performed to determine associations with gestational age for each domain, adjusting for infant sex, age at testing, and maternal education. Spearman correlations ( *rho*) and principal component analysis were used to investigate correlational structure across domains.

**Results:**

By parental report, gestational age at birth predicts language (standardised estimates 0.13 to 0.21, corrected *p*-values <0.05), but not attention or executive functions, at age 2 years. Scores across domains were modestly correlated: language and attention ( *rho* = 0.29 to 0.32; *p- *values <0.05), attention and executive functions ( *rho* = 0.19 to 0.30; *p- *values <0.05), language and executive functions ( *rho* = 0.19; *p- *values <0.05). The first principal component explained a substantial proportion of variance (35.4%) amongst measures, indicating a general factor.

**Conclusions:**

Parents of 2-year-old preterm children report reduced language ability, but similar attention and executive function development compared to term-born children. A general factor explains a significant proportion of variance in parental ratings of cognition across domains in early childhood.

## Plain Language Summary

Children born preterm often experience difficulties in a range of cognitive skills, typically assessed by an examiner. These include general cognitive abilities and particular areas such as language, attention and executive functions. There is a need for efficient, scalable assessments that are relevant to everyday behaviours. For example, parental questionnaires are increasingly used, but it is unclear how well they can detect early cognitive difficulties in preterm children. Cognitive skills are also often correlated, such that people who perform well in one area usually perform well in another area. However, it is unknown if parental ratings of different skills correlate similarly. We used parental questionnaires to assess the language, attention and executive functions of 2-year-olds (96 preterms and 87 terms). Parents of preterm toddlers report reduced language abilities but similar attention and executive functions compared to term-born children. We found that these three skills are correlated with an underlying general ability that links them based on parents’ ratings. Our findings provide insight into parents’ approach to distinguishing abilities in different domains. Future studies should compare parental reports with examiner-administered assessments to understand how both types of assessments relate to one another in early life.

## Introduction

Children born preterm are at risk of cognitive impairment extending across the life course ( Johnson & Marlow, 2017). Cognitive impairment is the most common neurodevelopmental sequelae of birth at less than 32 weeks’ gestation, surpassing rates of neurosensory disability and specific diagnoses such as autism, cerebral palsy and epilepsy ( Hirschberger *et al.*, 2018). Meta-analyses reveal that preterm children are at risk of deficits in general cognitive ability and in particular neuropsychological domains including language, attention and executive functions ( Allotey *et al.*, 2018; Arpi *et al.*, 2019; Barre *et al.*, 2011; Brydges *et al.*, 2018; Burstein *et al.*, 2021; Mulder *et al.*, 2009; van Houdt *et al.*, 2019; van Noort-van der Spek *et al.*, 2012). Although preterm children show large inter-individual variation in neuropsychological outcomes, there is strong evidence for a dose-dependent relation with degree of prematurity, in that children born earliest are most at risk for poor outcomes ( Anderson, 2014; Johnson, 2007).

Characterising cognition in the first few years of life is important for detecting developmental difficulties and for stratifying preterm infants at risk who may benefit from targeted early interventions. In several countries, neurodevelopmental assessment at 2 years of age is part of standard follow-up care for preterm infants, as this timepoint represents an important milestone for identifying major neurological and sensory disabilities ( Marlow, 2004; Seppänen *et al.*, 2024) and identifying toddlers which may benefit from additional support. Around 2 years of age, there is major growth in cognition and language, and this acquisition marks the transition from infancy to early childhood. Cognitive ability at 2 years is typically assessed in clinical and research settings using standardised examiner-administered developmental assessments, such as the Bayley Scales of Infant and Toddler Development-Fourth Edition (Bayley-4). In recent years, parent-report measures have gained traction to assess outcomes in both settings because of their high ecological value, relevance to adaptive behaviour in everyday settings, and because of cost-effectiveness and scalability ( Baker *et al.*, 2013; Johnson *et al.*, 2008, 2019; Loe *et al.*, 2015). Unlike examiner-administered assessments, parent-report measures index children’s abilities across multiple environments and everyday activities, reflecting functional skills that may not be apparent in structured testing. Parental questionnaires also mitigate the practical constraints of data collection, particularly for children with the most pronounced impairments who may be unable to fully engage with stringent testing protocols. For example, the Parent Report of Children’s Abilities-Revised (PARCA-R) provides a validated assessment of children’s cognitive and language development at 24–27 months ( Johnson *et al.*, 2019). It is useful as an endpoint in randomised controlled trials of interventions designed to improve outcomes after preterm birth ( Dargaville *et al.*, 2023; Moore *et al.*, 2023). For a more comprehensive view of a child’s functioning and to identify subtle and specific deficits, adopting a transdiagnostic neuropsychological approach assessing cognition across multiple domains is recommended ( Anderson, 2014; Astle *et al.*, 2022). Previous research has shown that parental report at 2 years can be used to identify transdomain clusters of neurodevelopment at scale, replicates across countries, has clinical validity and is useful for investigating upstream determinants of neurodevelopmental outcomes ( Haider *et al.*, 2025). Evidently, parent-report questionnaires with adequate psychometric properties may serve as scalable, complementary tools to labour- and time-intensive direct testing; but less is known about whether these measures of other domains relevant to preterm birth, including attention and executive function, capture differences in toddlerhood. Yet, to better understand the nature of impairments in children born preterm and avoid underestimating difficulties experienced in the early years, it is necessary to use instruments that measure performance in multiple domains.

Cognitive measures often share a correlational structure, whereby test scores across different domains tend to positively correlate. An underlying construct, commonly known as the general cognitive function or ‘ *g*’, captures this phenomenon of positive test score correlations. Its tendency to explain a substantial proportion (~30–40%) of test score covariances has been an exceptionally well-replicated finding in psychological science since Spearman’s initial report, and is invariant to test battery content and composition ( Carroll, 1993; Johnson *et al.*, 2004; Johnson *et al.*, 2008a; Spearman, 1904). There is some evidence for positive correlations (and thus, *g*), across measures in early life using a combination of parent-administered tests and reports of children’s cognitive abilities in twins ( Spinath *et al.*, 2003). However, the extent to which parent-report measures of language, attention and executive functions in young children are correlated is not known, but could offer new information about the nature of preterm birth-associated cognitive impairment as rated by parents.

In a cohort of toddlers enriched for preterm birth, we investigated associations between gestational age (GA) and language, attention and executive functions at 2 years using parent-report measures. Our hypotheses were that 1) low GA predicts poorer outcomes at 2 years in language, attention or executive functions, or combinations of these; 2) measures within and across these domains are positively correlated; and 3) a factor of general cognitive ability, *g*, can be derived from parent-report measures, suggesting consistent individual differences across language, attention and executive functions at age 2 years.

## Methods

The STROBE guidelines were used for reporting of this observational study (see Extended data).

### Study sample

Preterm infants (GA ≤ 33 weeks) and a comparator group GA >36 weeks were recruited between 2016 and 2021 to the Theirworld Edinburgh Birth Cohort (TEBC), a prospective, longitudinal study designed to investigate the impact of preterm birth on neurodevelopment ( Boardman *et al.*, 2020). Exclusion criteria were major congenital malformations, chromosomal abnormalities, congenital infection, parenchymal brain injury (cystic periventricular leukomalacia, haemorrhagic parenchymal infarction or post-haemorrhagic ventricular dilation). As such, the cohort is representative of the majority of survivors of modern neonatal intensive care practices ( Boardman *et al.*, 2020; Sullivan *et al.*, 2025). Ethical approval was obtained from the National Research Ethics Service, South East Scotland Research Ethics Committee (16/SS/0154, dated 5/10/2016). Written informed consent was obtained from a person with parental responsibility for each participant. Infants were invited for assessment at 2 years of age. We included all children who attended 2-year follow-up and had at least one parent-report measure available in any of the domains of language, attention or executive functions (N = 183).

### Clinical and demographic measures

Clinical and demographic data were collected through questionnaires administered to the primary caregiver and review of medical records. Participant characteristics are reported in Table 1. Neonatal co-morbidities were defined as previously described ( Sullivan *et al.*, 2020): sepsis was defined as blood culture positive for pathogenic organism (culture positive) or clinically suspected infection treated with intravenous antibiotics for ≥5 days (culture negative); necrotising enterocolitis was defined as medical treatment for ≥7 days or surgical treatment; bronchopulmonary dysplasia was defined as the need for supplemental oxygen therapy or respiratory support at 36 weeks corrected gestational age; retinopathy of prematurity was defined as requiring treatment with laser therapy. Socioeconomic status was measured with the Scottish Index of Multiple Deprivation 2016 (SIMD) and maternal education (highest final educational qualification). SIMD is derived from a family’s postal code at birth, which uses multiple dimensions to assign a level of deprivation to the neighbourhood, ranked from most (1) to least (6976) deprived ( Scottish Index of Multiple Deprivation 2016). To use SIMD rank, which is subject to Crown Copyright, no further permission was required as users can copy, publish, distribute, transmit, adapt and exploit this variable providing they acknowledge the source of information. Maternal smoking during pregnancy was self-reported as cigarette smoking status at time of booking the pregnancy and is coded as a binary variable (Yes: current or No: never/previous). Maternal alcohol use during pregnancy was self-reported as a binary variable (Yes: any alcohol consumed or No: no alcohol consumed).

### Neuropsychological outcome measures

At two years of age, families participated in a comprehensive assessment of child neurodevelopment as described in the study protocol ( Boardman *et al.*, 2020). The parent/carer-report measures were collected for the following domains:

***Language.*** The Vineland Adaptive Behaviour Scales (VABS)-Third Edition Comprehensive Parent/Caregiver Form was completed. The questionnaire produces a communication composite standard score with mean of 100 and standard deviation (SD) of 15, and scaled scores of Receptive Communication and Expressive Communication, with mean of 15 and SD of 3. VABS is a norm-referenced measure of adaptive behaviour, and communication scores provide information about language proficiency of individuals in the context of their daily environment ( Sparrow *et al.*, 2005). Evidence of concurrent and predictive validity of VABS in preterm children has been previously demonstrated ( Rosenbaum *et al.*, 1995). The VABS instrument is a proprietary material, purchased from Pearson Assessments and is used under their standard licensing agreement for research purposes. Due to copyright restrictions, the VABS cannot be reproduced or included in this manuscript. Parent-report of language was also derived from MacArthur-Bates Communicative Development Inventory Words and Sentences (CDI-WS) ( Fenson *et al.*, 2006), using scores of Word Production. CDI-WS Word Production score is generated from a vocabulary production checklist, organised into 22 semantic categories. CDI-WS is designed for children 16–30 months. The CDI forms are proprietary materials, purchased from Brookes Publishing and are used in accordance with their end user licensing agreement. Due to copyright restrictions, the CDI cannot be reproduced or included in this manuscript.

***Attention.*** Parent-reported attention was acquired from scores of Attentional Shifting and Attentional Focusing, using the Early Childhood Behaviour Questionnaire (ECBQ), a temperament questionnaire designed for children 18–36 months ( Putnam *et al.*, 2006). The ECBQ is freely available for non-commercial research use upon request from the original authors and cannot be publicly posted due to distribution guidelines ( https://research.bowdoin.edu/rothbart-temperament-questionnaires/instrument-descriptions/the-early-childhood-behavior-questionnaire/).

***Executive Functions.*** Executive functions were assessed using the Behaviour Rating Inventory of Executive Function-Preschool version (BRIEF-P) ( Gioia *et al.*, 1996). The standardisation sample comprised various clinical groups, including preterm children. Scores were derived for BRIEF-P subscales: Inhibit, Shift, Working Memory, Emotional Control, Plan/Organise, as well as the General Executive Composite (GEC). GEC and each subscale are age- and sex-normed to T-scores, wherein median is 50 and a score above 65 is recognised as clinically significant. Inconsistency and Negativity scores are also determined to assess validity of parent ratings, and elevated scores were removed. The BRIEF-P is proprietary material, administered in accordance with terms of use set forth by Psychological Assessment Resources (PAR, Inc.) and is used with permission under their licensing agreement for research purposes. Due to copyright restrictions, the BRIEF-P cannot be reproduced or included in this manuscript.

### Statistical analyses

All statistical analyses were performed in R (version 4.2.3) ( https://www.r-project.org/).

***Participant characteristics.*** Maternal and infant demographic and clinical characteristics were compared for preterm and term infants in Table 1. Standardised mean difference (Cohen’s *d*) was used to quantify effect sizes, with 0.2, 0.5, and 0.8 indicating small, medium, and large effect sizes, respectively ( Cohen, 1992).

***Associations between gestational age and neuropsychological outcomes.*** As outcome variables were not normally distributed, Mann-Whitney *U- *tests were used to assess differences in distributions of cognitive outcomes for preterm and term children at 2 years. Linear regression models were used to investigate the association between gestational age (GA) at birth and cognitive outcomes at 2 years. The baseline model was each outcome measure (dependent variable) regressed on GA at birth only (predictor variable). The adjusted model included the following covariates, selected because of their associations with cognition or brain development in early childhood ( Benavente-Fernández *et al.*, 2019; Durrant *et al.*, 2020; Loeb *et al.*, 2020; Mckinnon *et al.*, 2023; Sanchez *et al.*, 2019): infant sex, family-level socioeconomic status operationalised as maternal education, and age at testing. Reported *p- *values are adjusted for the false discovery rate ( *p _FDR_*) using the Benjamini-Hochberg procedure, with the family of experiments being each domain. Multiple comparisons were corrected for in baseline and adjusted models separately, and significance was deemed *p*_FDR_ < 0.05 ( Benjamini & Hochberg, 1995).

***Correlational structure of neuropsychological outcome and principal component analysis to derive general factor of cognitive ability.*** Relationships between neuropsychological outcome scores were assessed using Spearman’s correlations. BRIEF-P scores were reverse-coded for these analyses such that higher scores represent improved outcomes, in keeping with the direction of measurement of variables across the other domains. Principal component analysis (PCA) was used to examine the correlational structure of neuropsychological outcomes and quantify the proportion of shared variance between measures. The ten neuropsychological outcome scores, excluding composite scores, for individuals with complete data across all domains (n = 97) were first standardised to have zero mean and unit variance before applying PCA. Scores derived from the first unrotated principal component were extracted as an index of a general factor (g) of parent-reported ability. PCA was also repeated using varimax rotation to extract 3 components, based upon inspection of the scree plot for an inflection point and components with eigenvalues >1. Spearman’s correlation coefficient was also determined to assess relationship between unrotated PC1 scores and GA at birth. Welch’s t-test was also applied to assess differences in PC1 scores between preterm and term-children. Finally, we used multivariable linear regression to examine the association between birth GA and PC1 scores, with covariates infant sex, maternal education and age at testing.

## Results

### Participant characteristics

The study sample consisted of 183 mother and child dyads (96 preterm and 87 term-born children), characteristics of whom are shown in Table 1. Median GA at birth of the preterm group was 29.57 weeks and of the term group was 39.71 weeks. Socioeconomic status differed between groups, with more mothers of term-born children tending to have higher educational qualifications and lower socioeconomic deprivation, indicated by higher SIMD rank.

**Table 1.** Demographic and clinical characteristics of the participants.

	Preterm (n = 96)	Term (n = 87)	Effect size
**Child**			
Sex, n (%)			0.07
Male	54 (56.2)	46 (52.9)	
Female	42 (43.8)	41 (47.1)	
Gestational age/weeks, median (range)	29.57 (24.00–32.43)	39.71 (36.43–42.14)	6.01
Age at assessment/months ^a^, median (range)	24.0 (23.0–28.4)	23.9 (22.9–25.4)	0.20
Birthweight/g, median (range)	1295 (562–2160)	3520 (2410–4560)	5.53
Birthweight z–score/median (range)	0.23 (−3.04–2.04)	0.53 (−2.30–2.57)	0.49
Sepsis, n (%)	24 (25.0)	NA	NA
NEC, n (%)	1 (1.0)	NA	NA
Retinopathy of prematurity, n (%)	2 (2.1)	NA	NA
Bronchopulmonary dysplasia, n (%)	26 (27.7)	NA	NA
**Maternal/antenatal**			
Maternal age/years, median (range)	33.50 (21.00–43.00)	34.00 (24.00–47.00)	0.34
Alcohol use during pregnancy, n (%)	4 (4.2)	3 (3.4)	0.04
Smoked during pregnancy, n (%)	5 (5.3)	2 (2.3)	0.04
Gestational diabetes, n (%)	2 (2.1)	4 (4.6)	0.14
Preeclampsia, n (%)	19 (19.8)	5 (5.7)	0.43
Antenatal corticosteroid administration, n (%)	90 (93.8)	NA	NA
Antenatal MgSO _4_ for fetal neuroprotection, n (%)	71 (74.0)	NA	NA
Mother highest educational qualification, n (%)			0.65
Basic high school qualification	9 (9.5)	2 (2.3)	
Advanced high school qualification	4 (4.2)	1 (1.1)	
College qualification	20 (21.1)	8 (9.2)	
University undergraduate degree	28 (29.5)	42 (48.3)	
University postgraduate degree	31 (32.6)	34 (39.1)	
Not applicable	3 (3.2)	0 (0.0)	
SIMD rank, median (range)	4674 (370–6966)	5480 (727–6967)	0.33

Missing data: Age at assessment was not available for n = 3 preterm children; Smoked during pregnancy was not available for n = 1 preterm children; Mother highest educational qualification was not available for n = 1 preterm children; SIMD rank was not available for n = 17 preterm children and n = 5 term children.

^a^ Corrected for prematurity in the preterm group. SIMD = Scottish Index of Multiple Deprivation 2016.

### Preterm and term group differences in neuropsychological outcome at 2 years

**Figure 1.**
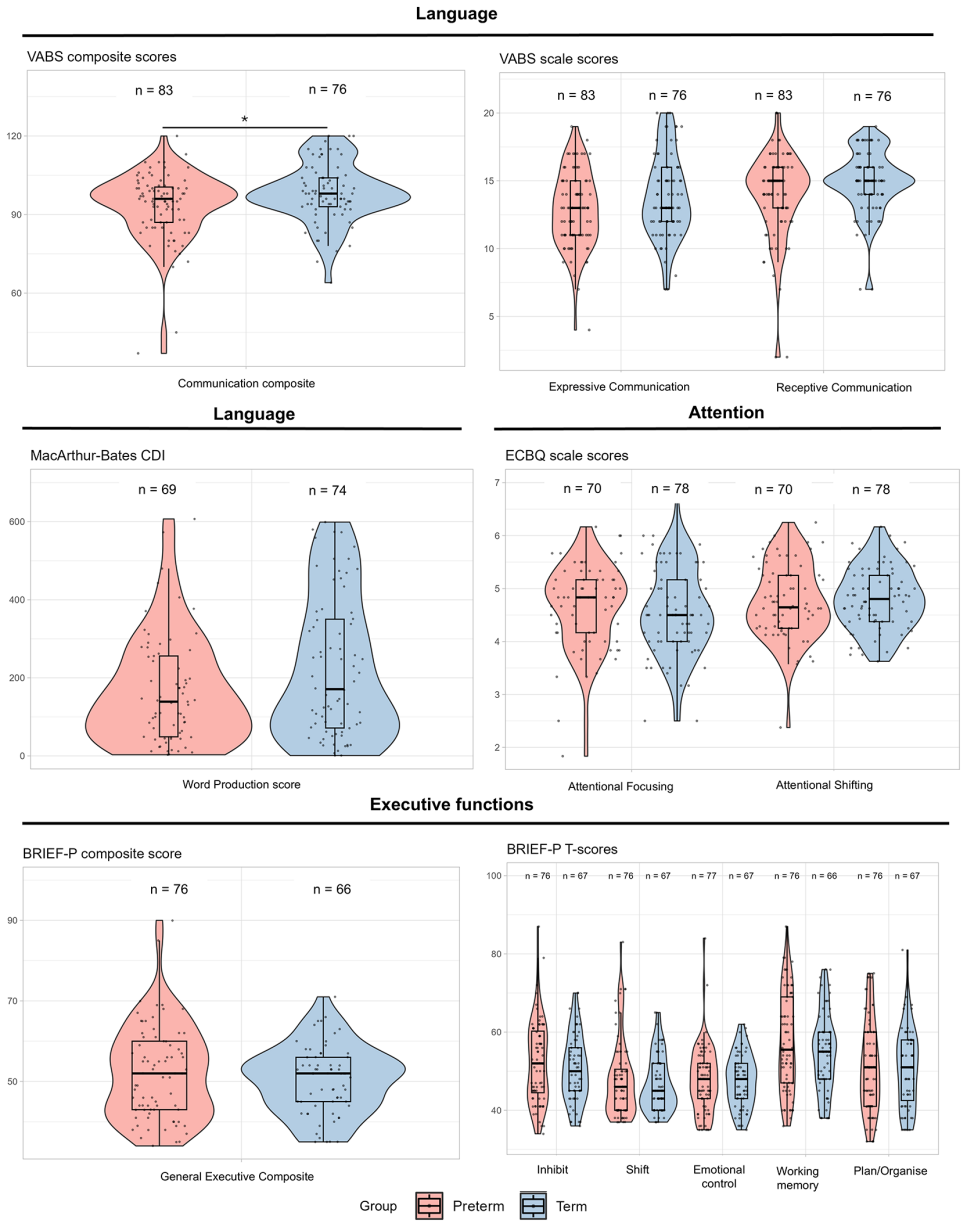
Group-wise analysis of parent-report measures across domains at 2 years. Violin and box plots of scores of language, attention and executive functions from parent-completed questionnaires for preterm and term-born children at 2 years. *p < 0.05.

The median VABS communication composite score at 2 years was significantly lower in preterm children compared to term children, but the magnitude of difference was small: 96 (IQR 87 to 101) versus 98 (IQR 93 to 104), *p* = 0.04, *d* = −0.41 ( Figure 1, Table 2). There were no statistically significant groupwise differences in the VABS sub-scales of receptive and expressive communication, or in CDI word production ( *d* = −0.32 to −0.36, all *p*-values >0.05; Figure 1, Table 2). On measures of attention derived from the ECBQ and measures of executive function derived from scales of the BRIEF-P, there were no statistically significant differences between preterm and term-born children (all *p*-values >0.05).

**Table 2.** Median scores from parent-report measures between preterm and term-born children at 2 years. Mann-Whitney *U- *tests were used to assess differences in distributions of cognitive outcomes for preterm and term children at 2 years. Statistically significant differences are highlighted in bold, where *p* < 0.05.

Outcome measure	Preterm		Term		*p-*value	Effect size
	N	M (IQR)	N	M (IQR)		
**Language**						
VABS Communication Composite	83	96 (87, 101)	76	98 (93, 104)	**0.04**	−0.41
VABS Receptive Communication	83	15 (13, 16)	76	15 (14, 16)	0.06	−0.35
VABS Expressive Communication	83	13 (11, 15)	76	13 (12, 16)	0.08	−0.32
MacArthur-Bates CDI Word Production	69	139 (49, 256)	74	171 (72, 350)	0.11	−0.36
**Attention**						
ECBQ Attentional Focusing	70	4.83 (4.17, 5.17)	78	4.50 (4.00, 5.17)	0.30	0.13
ECBQ Attentional Shifting	70	4.65 (4.25, 5.26)	78	4.80 (4.38, 5.25)	0.60	−0.12
**Executive Function**						
BRIEF-P General Executive Composite	76	52 (43, 60)	66	52 (45, 56)	0.60	0.16
BRIEF-P Inhibit	76	52 (45, 60)	67	50 (45, 56)	0.40	0.19
BRIEF-P Shift	76	46 (40, 51)	67	45 (40, 52)	0.80	0.16
BRIEF-P Emotional control	77	48 (43, 52)	67	48 (43, 52)	0.90	0.02
BRIEF-P Working memory	76	56 (47, 69)	66	55 (48, 60)	0.30	0.22
BRIEF-P Plan/Organise	76	51 (41, 60)	67	51 (43, 58)	0.90	0.05

### Associations between gestational age at birth and neuropsychological outcome

**Figure 2.**
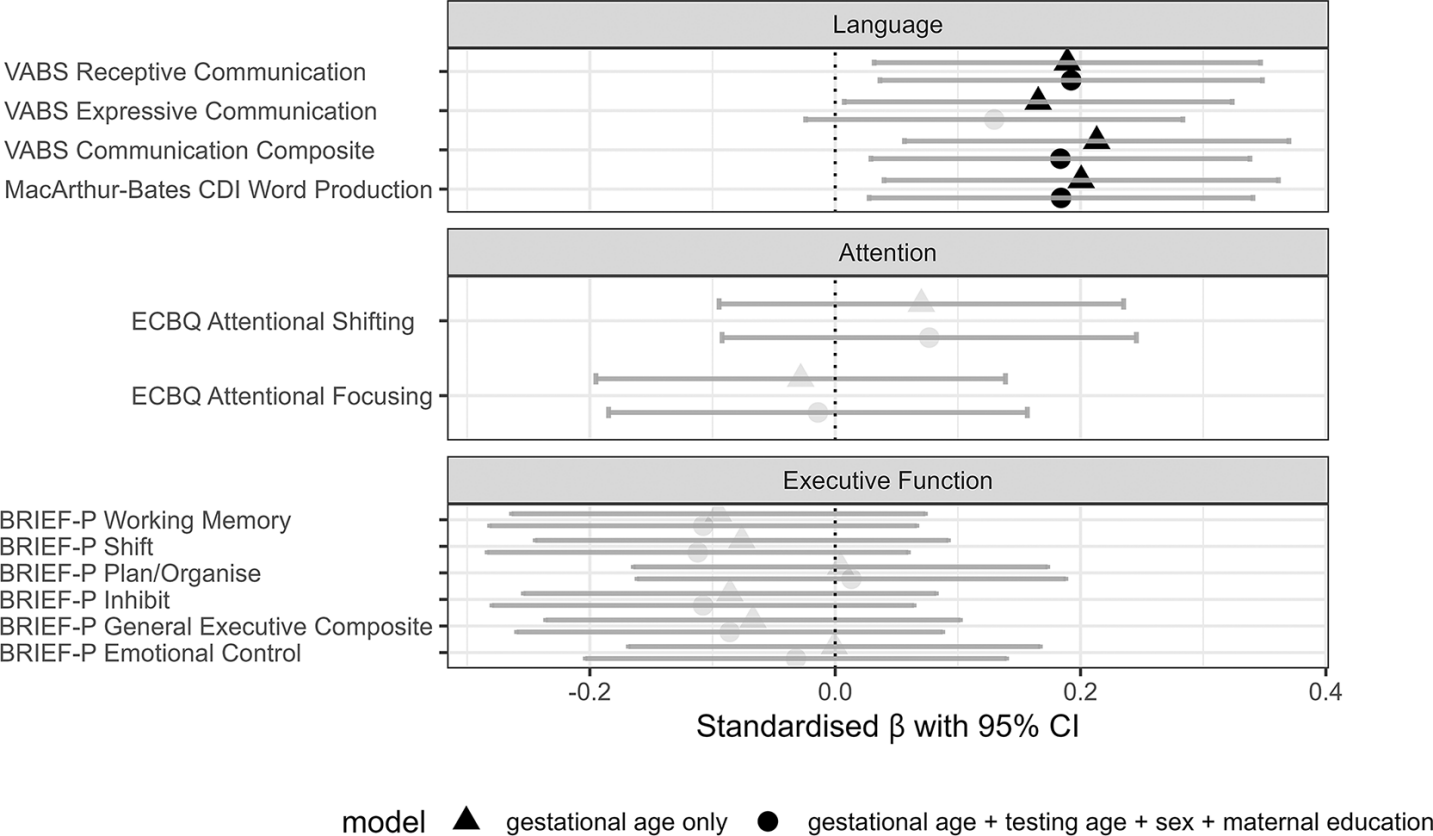
Associations between gestational age at birth and parent-report measures at 2 years. Points show standardised effect size (regression coefficients) and 95% confidence intervals for associations. Baseline models (triangle) regress gestational age at birth on outcome, and adjusted models (circle) additionally covary for age at testing, infant sex and maternal education. Significant associations are denoted by dark filled shapes, where *p* < 0.05 (FDR corrected for multiple comparisons within a domain).

Associations between GA at birth and language, attention and executive function outcomes at 2 years were analysed by linear regression ( Figure 2, Table 3). In unadjusted models, GA at birth positively associated with language at 2 years, with later GA predicting higher scores of expressive (β = 0.165, SE 0.081, *p*_FDR_ = 0.042), receptive (β = 0.189, SE 0.080, *p*_FDR_ = 0.026) and composite communication (β = 0.213, SE 0.080, *p*_FDR_ = 0.026) on VABS and CDI word production (β = 0.201, SE 0.082, *p*_FDR_ = 0.026). In models adjusted for sex, age at testing, and maternal education, a similar pattern of results was observed, except that the association between GA and VABS expressive communication was no longer statistically significant (β = 0.130, SE 0.078, *p*_FDR_ = 0.100). Post-hoc analyses revealed that this attenuation was driven by sex (β = −0.526, SE 0.078, p = 0.008), which accounted for an additional 9.9% of variance in VABS expressive communication, whereas maternal education and age at testing did not significantly contribute. There were no significant associations between GA and attention or executive functions (β = |0.001 to 0.112|, SE 0.084 to 0.089, all *p- *values >0.05).

**Table 3.** Associations between gestational age at birth and 2-year parent-report measures, adjusting for covariates. Standardised betas (β), standard errors and *p* values (raw and adjusted for false discovery rate, *p*_FDR_, using Benjamini-Hochberg procedure) are reported from regression models where gestational age at birth is regressed onto neuropsychological outcome measures. Baseline models regress gestational age at birth on outcome, and adjusted models additionally covary for age at testing (corrected for prematurity), sex and maternal education. Bold text denotes *p* < 0.05.

Outcome measure	n	model	β	SE	*p*-value (raw)	*q-*value ( *p*_**FDR**_)	Multiple R ^**2**^	Adjusted R ^**2**^
VABS Communication Composite	156	baseline	0.213	0.080	**0.008**	**0.026**	0.044	0.038
		adjusted	0.184	0.079	**0.021**	**0.030**	0.134	0.111
VABS receptive communication	156	baseline	0.189	0.080	**0.020**	**0.026**	0.035	0.029
		adjusted	0.192	0.080	**0.017**	**0.030**	0.118	0.095
VABS expressive communication	156	baseline	0.165	0.081	**0.042**	**0.042**	0.027	0.020
		adjusted	0.130	0.078	0.100	0.100	0.142	0.119
CDI word production	143	baseline	0.201	0.082	**0.016**	**0.026**	0.041	0.034
		adjusted	0.184	0.080	**0.023**	**0.030**	0.169	0.145
ECBQ attentional focusing	145	baseline	−0.028	0.085	0.742	0.742	0.001	−0.006
		adjusted	−0.014	0.087	0.872	0.872	0.021	−0.007
ECBQ attentional shifting	147	baseline	0.070	0.084	0.405	0.742	0.005	−0.002
		adjusted	0.077	0.086	0.375	0.750	0.021	−0.006
BRIEF-P General executive composite	139	baseline	−0.067	0.086	0.439	0.658	0.004	−0.003
		adjusted	−0.086	0.089	0.334	0.500	0.020	−0.009
BRIEF-P shift	140	baseline	−0.076	0.086	0.377	0.658	0.006	−0.002
		adjusted	−0.112	0.088	0.203	0.456	0.039	0.011
BRIEF-P inhibit	140	baseline	−0.086	0.086	0.319	0.658	0.007	0.000
		adjusted	−0.108	0.088	0.222	0.456	0.035	0.006
BRIEF-P emotional control	141	baseline	−0.001	0.086	0.993	0.993	0.000	−0.007
		adjusted	−0.032	0.088	0.716	0.859	0.028	−0.001
BRIEF-P working memory	139	baseline	−0.095	0.086	0.271	0.658	0.009	0.002
		adjusted	−0.108	0.089	0.228	0.456	0.015	−0.015
BRIEF-P plan/organise	140	baseline	0.004	0.086	0.960	0.993	0.000	−0.007
		adjusted	0.013	0.089	0.882	9.882	0.004	−0.026

### Correlational structure of neuropsychological outcome measures at 2 years and principal component analysis

**Figure 3.**
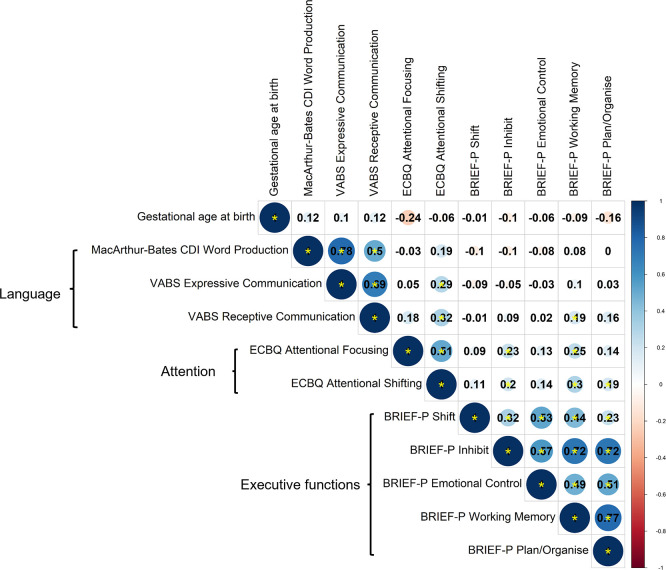
Correlations between parent-report measures across domains, assessed by Spearman’s correlation for complete observations (n = 97). Correlation coefficients are shown. Colour scale shows magnitude and direction of correlation coefficients. Statistically significant correlations are denoted by a yellow asterisk, whereby *p* < 0.05.

**Figure 4.**
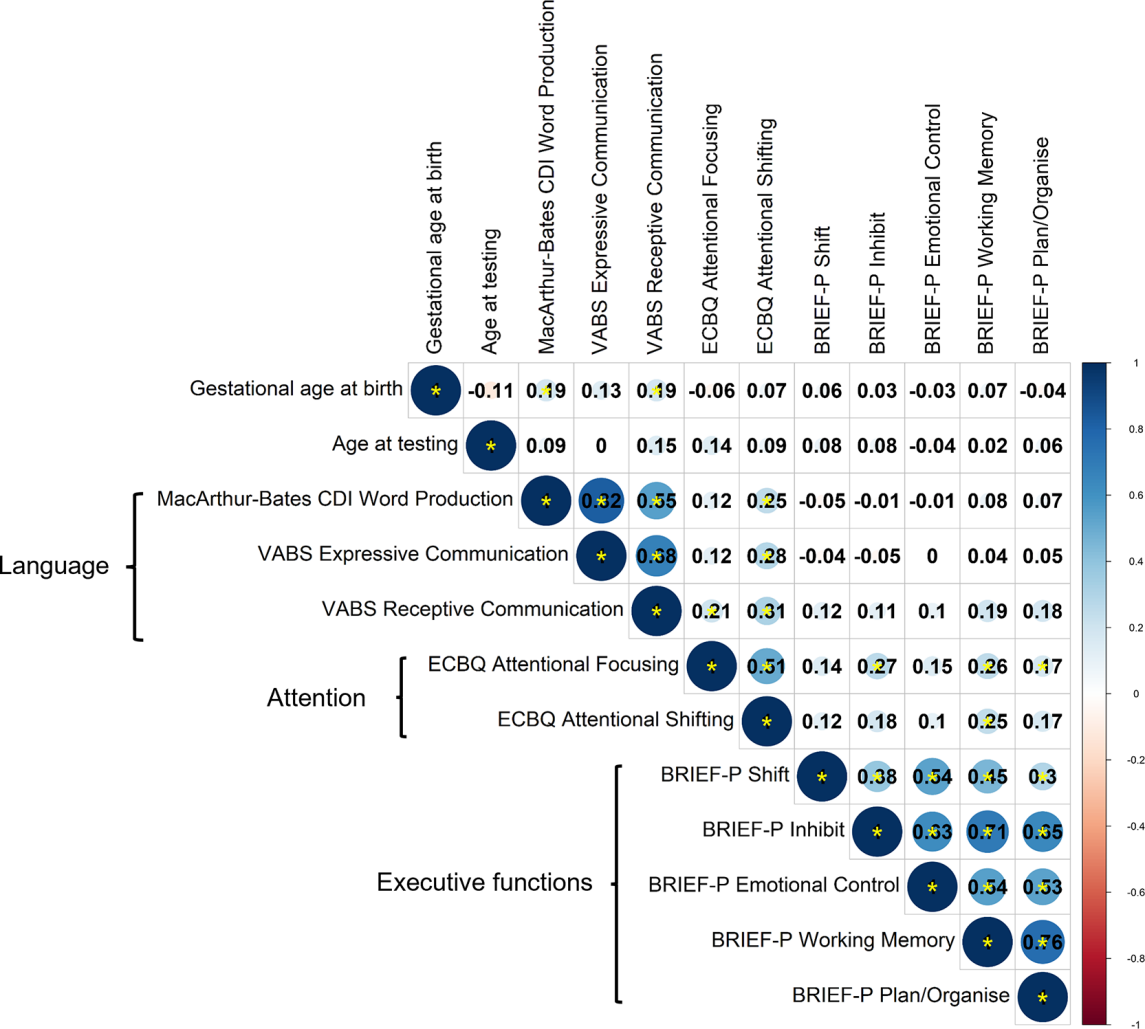
Correlations between parent-report measures across domains, assessed by Spearman’s correlation for pairwise observations (maximum n = 148). Correlation coefficients are shown. Colour scale shows magnitude and direction of correlation coefficients. Statistically significant correlations are denoted by a yellow asterisk, whereby *p* < 0.05.

Correlations between language, attention and executive function measures were assessed by Spearman’s correlation coefficient, *rho,* using data from 97 participants with complete observations ( Figure 3). A smaller sample size was used for these analyses due to availability of complete data, but analyses of pairwise correlations for the full sample produced a similar pattern of correlations, suggesting generalisability to the wider sample ( Figure 4). Given the lack of differences between preterm and term toddlers in most measures, both preterm and term toddlers were included in analyses.

Correlations within instrument and within outcome domain were variable; namely, between language measures derived from VABS and CDI word production ( *rho =* 0.50 to 0.78); between ECBQ attentional focusing and shifting ( *rho* = 0.51); and between BRIEF-P subscales ( *rho =* 0.23 to 0.77). Most tests across domains were significantly correlated, with weak effect sizes ( *rho =* 0.19 to 0.32). Importantly, no measures significantly correlated with age at testing in the whole sample, suggesting that testing age is not driving correlations across measures ( Figure 4).

Using PCA, we derived an underlying factor for parent report measures at 2 years. The first principal component (PC1) explained 35.4% of variance across measures ( Table 4). BRIEF-P measures more strongly indicate PC1 with higher loadings (0.56 to 0.88) compared to attention (0.40 to 0.53) and language (0.11 to 0.34) measures ( Table 4). Despite the uneven distribution of PC1 loadings, all but two cognitive tests (CDI Word Production and VABS receptive communication) had loadings >0.3 on PC1, which is standard in psychometrics to demonstrate a significant contribution ( Hair, 1998). The unrotated PC1 scores were not correlated with gestational age at birth ( *rho* = 0.12, *p* = 0.23) and did not differ between preterm and term-born children in groupwise analyses (t(90.9) = 0.27, p = 0.79). In a multivariable linear regression, we also observed no significant association of PC1 scores with gestational age at birth (β < 0.001, SE 0.101, p = 0.998), sex (male: β = −0.147, SE 0.206, p = 0.479), maternal education (university-level or higher: β = 0.349, SE 0.253, p = 0.170) or age at testing (β = −0.085, SE 0.105, p = 0.421).

**Table 4.** Principal component analysis loadings for each measure. Loadings for the first unrotated principal component and varimax-rotated components derived from the 2-year parent-report measures. Loadings >0.3 are shown in bold.

Outcome measure	PC1 unrotated	Rotated components		
		PC1	PC2	PC3
CDI Word Production	0.11	−0.07	**0.88**	−0.07
VABS receptive communication	0.22	−0.04	**0.93**	0.11
VABS expressive communication	**0.34**	0.09	**0.82**	0.21
ECBQ attentional focusing	**0.40**	0.08	−0.04	**0.90**
ECBQ attentional shifting	**0.53**	0.21	0.27	**0.77**
BRIEF-P shift	**0.56**	**0.64**	−0.11	0.02
BRIEF-P inhibit	**0.83**	**0.85**	−0.03	0.19
BRIEF-P emotional control	**0.72**	**0.81**	−0.07	0.01
BRIEF-P working memory	**0.88**	**0.84**	0.19	0.21
BRIEF-P plan/organise	**0.80**	**0.83**	0.09	0.10
Eigenvalue	3.54	3.54	2.48	1.20
Variance explained (%)	35.4	32.3	24.3	15.6
Cumulative variance (%)	-	32.3	56.6	72.3

**Figure 5.**
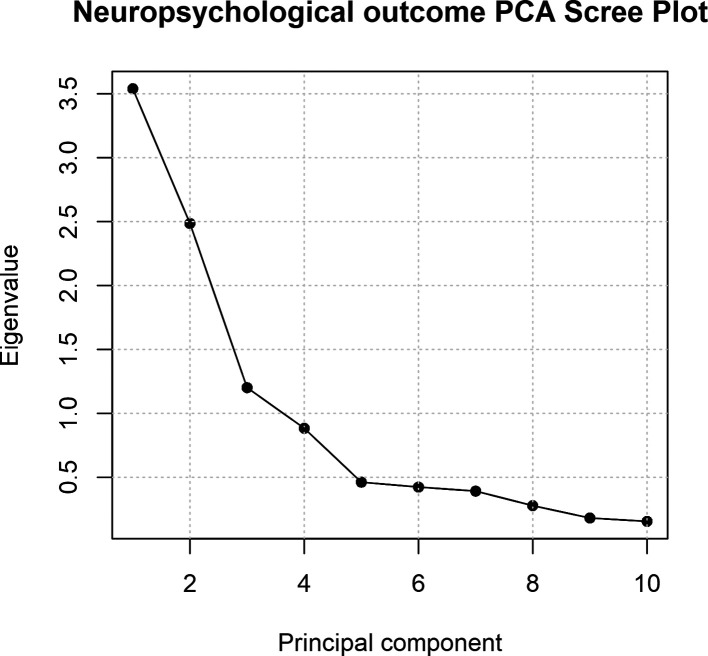
Scree plot for the principal component analysis (unrotated). Eigenvalues are plotted against the number of principal components derived from parent-report measures.

Further, the scree plot indicated three factors with eigenvalues >1; these three factors were extracted using varimax rotation and cumulatively explained 72.3% of variance ( Figure 5, Table 4). Extracting three rotated components yielded a loading pattern mainly consistent with the measurement instruments: PC1 showed preferential loadings from BRIEF-P scores (executive functions), PC2 VABS and CDI (language); PC3 on the two ECBQ attentional scores.

## Discussion

In a sample of 2-year-olds that included preterm and term-born children, we assessed language, attention and executive functions using parental ratings. We found that low birth GA predicts poorer language outcomes at age 2 years; there was no evidence for similar effects for attention and executive functions. Neuropsychological outcome measures were modestly correlated across all domains. PCA revealed three components, cumulatively capturing 72.3% of variance, with strong loadings for measures specific to each domain, mostly within measurement instrument. Although these parent-report measurements can be decomposed into separable domains, the first unrotated component captured a substantial minority of variance among measures from all three domains (35.4%), suggesting a domain-general factor underlying parental ratings. This factor may reflect general cognitive ability, *g.* Another (not mutually exclusive) possibility is that this factor reflects parents’ general impression of their child’s development and cognitive abilities. Despite consistent individual differences, the absence of an association between gestational age and the general measure of parent-reported cognitive function further indicated that preterm-related associations with cognitive function were not pervasive across all domains.

Evidence for an association between lower GA at birth and poorer parent-rated language is consistent with previous research with this age group. Language differences following preterm birth have been described between 2 and 12 years, for both rating and performance-based measures, with comparable effect sizes to our findings (for meta-analyses, see Barre *et al.*, 2011; van Noort-van der Spek *et al.*, 2012). The effect of GA at birth on language outcome is dose-dependent, as previously demonstrated in this cohort at 2 years with measures strongly correlated with GA at birth ( Valavani *et al.*, 2022). The association between birth GA and expressive language was not statistically significant when adjusting for sex, consistent with previous research showing that male sex is strongly predictive of poorer language and general cognitive outcomes in children at 2 years of age ( Haider *et al.*, 2025; Linsell *et al.*, 2015; O’Driscoll *et al.*, 2018). Importantly, we note large variability in effect sizes across all domains, possibly owing to other demographic, psychosocial, genetic or environmental factors ( Stålnacke *et al.*, 2015).

Consistent with the lack of evidence for a relationship between attention and birth GA we observed, de Jong *et al.* (2015) reported no differences between preterm and term toddlers of similar sample sizes on parent-completed ECBQ attentional shifting and attentional focusing scales, despite observing differences on eye-tracking measures of attention. Other cohort studies of smaller samples have reported differences in attentional focusing, but not attentional shifting, between 18–36 months ( Cosentino-Rocha *et al.*, 2014; Lejeune *et al.*, 2015). This discrepancy may point to potentially stronger sensitivity of parental report to attentional focusing compared to shifting, and of direct measures of attention compared to parental report. Meta-analyses of direct measures of attention at 2 years reveal poorer performance on a wide range of tasks ( Arpi *et al.*, 2019; Burstein *et al.*, 2021), though to our knowledge, no similar collated analysis of rating-based measures of attention at this age has been reported. Additional research is therefore necessary to understand the difference between direct and parent-reported measures of attention, and the nature of attention differences as a function of preterm birth.

Evidence from parental report studies of executive function difficulties for preterm children is also inconsistent, with studies describing difficulties in all ( Loe *et al.*, 2015), some ( Baron *et al.*, 2011; Blasco *et al.*, 2024) and no BRIEF-P sub-scales of executive functions ( O’Meagher *et al.*, 2017) at preschool age (3–5 years). Questionnaire ratings of executive functions at 2 years, apart from BRIEF-P, are sparse due to only being recently developed and limited validation of instruments in the youngest age groups ( Hendry & Holmboe, 2021; Nilsen *et al.*, 2017), but task-based assessments reveal poorer performance of preterm toddlers in domains particularly related to cognitive flexibility (such as shifting) ( Pozzetti *et al.*, 2014; Sandoval *et al.*, 2022; Van Den Brande *et al.*, 2024). As such, while measurable differences between preterm and term toddlers may be reported in early life, these differences in executive function and attention may only become apparent in day-to-day behaviour in childhood. Parents may thus detect these differences with time, as cognitive demands increase and/or as comparison to peers becomes possible. Indeed, the effect size of parent-reported group differences increases with age for language, attention and executive functions ( Allotey *et al.*, 2018; Burstein *et al.*, 2021; van Noort-van der Spek *et al.*, 2012). Interestingly, group differences have been reported by teachers, but not parents, on measures of both attention and executive function at 4–5 years ( O’Meagher *et al.*, 2017; O’Meagher *et al.*, 2020; Sejer *et al.*, 2019), suggesting differing insights into domains of cognition depending on the relationship between rater and child, context or expertise.

As higher-order processes required for goal-directed behaviour, attention and executive functions are separable but interrelated constructs. The development of attentional skills overlaps with that of executive functioning in the early years, with a core literature arguing that the maturation of attentional capacity contributes to various emerging executive functions ( Garon *et al.*, 2008; Reynolds & Romano, 2016). In the present study, significant cross-domain correlations were observed, particularly between ECBQ attentional measures and BRIEF-P Working memory and Inhibit, consistent with this developmental interdependence. Previous research in both preterm and typically developing children shows that some individuals with low working memory performance are frequently rated by teachers and parents as having elevated attention problems ( Aarnoudse-Moens *et al.*, 2013; Gathercole *et al.*, 2008). Furthermore, de Kievet et al. (2012) showed that working memory mediates the relationship between birth GA and inattentive behaviours at school age. At the same time, the rotated PCA solution identified distinct components corresponding to each domain, in line with established two-factor models of attentional control and executive function that sample multiple subdomains of these processes in early childhood ( Deodhar & Bertenthal, 2023). Together, these findings suggest that while attention and executive functions are closely related, they retain measurable differentiation at 2 years of age.

Importantly, while no associations were observed between birth GA and attention and executive functions, correlations were present across all three domains and substantial shared variance was accounted for across measures. In terms of general cognition, *g* typically accounts for approximately 30–40% of the variance among test scores, shows relatively even loadings across domains, and is invariant to cognitive test battery materials and content provided a broad set of cognitive domains is sampled ( Deary *et al.*, 2010; Johnson *et al.*, 2004; Johnson *et al.*, 2008b; Neisser *et al.*, 1996). Given that our first factor (unrotated PC1) explained a similar proportion of variance (35.4%), with measures from all domains showing significant loadings, we provide evidence that a general factor *, g*, can be derived from parental report measures. Part of this factor may reflect shared method variance inherent to parental ratings, such as parents’ general impressions of their child resulting in uniform responding. However, PC1 was not limited to a single instrument and included measures spanning all three domains, indicating that it is unlikely to be purely method-based. Furthermore, questionnaires with items rating behaviours thought to be external expressions of cognitive abilities have demonstrated concurrent validity with task-based cognitive measures ( Johnson *et al.*, 2008b; Waschbusch *et al.*, 2000), supporting the interpretation that the observed domain-general factor does not capture solely general parental impressions. Notably, the domain-general factor was not associated with birth GA, infant sex or maternal education, suggesting that the effects of prematurity and demographic influences are not pervasive across parent-reported cognitive domains at 2 years.

Although birth GA was associated with language outcomes, language measures contributed relatively weakly to the general factor. Specifically, VABS expressive communication, while meeting criteria for inclusion in PC1, showed the lowest loading and was also the only language measure for which association with birth GA was attenuated after adjusting for covariates. This overlap suggests that while some variance in this measure is accounted for by the domain-general factor, other variance may reflect demographic influences, and that GA-related language differences are unlikely to be the primary drivers of the domain-general factor.

Examination of the rotated PCs clarifies domain-specific contributions. One component primarily reflected executive functions (all measured via BRIEF-P), another language (measured across VABS and CDI) and a third attention (both ECBQ subscales). For the language factor, the alignment of measures from different instruments suggests that this component captures a coherent developmental domain rather than being an artefact of a single questionnaire. That unique measures of a single domain (specifically VABS and CDI for language) showed similarly strong loadings for a specific rotated PC indicates that similar constructs are “mapping” to specific components, beyond those from a single assessment tool. Although the executive function and attention components were derived from subscales within single instruments, the pattern of loadings was consistent with theoretically defined domains ( Deodhar & Bertenthal, 2023), supporting the interpretation that the solution reflects meaningful developmental constructs rather than purely instrument-specific variance. The uneven distribution of loadings across domains may relate to strong intercorrelations within particular domains in this age group or in relation to prematurity, as well as more available measures for particular domains (e.g. BRIEF-P for executive functions) than others (e.g. attention).

Beyond reflecting the instruments included, the 3-component solution has developmental implications. By 2 years of age, language, attention and executive functions appear measurably differentiated in parent-report data, while still sharing substantial variance suggestive of general cognitive processes. Together, these findings suggest that while parent report reflects consistent individual differences across domains, it does not reflect the impact of prematurity in driving differences in domains beyond language. Nonetheless, as children who show difficulties in one domain are more likely to experience difficulties in others and generalised cognitive difficulties on direct measures are well-reported in the literature ( Anderson, 2014; Brydges *et al.*, 2018; Mulder *et al.*, 2009; Twilhaar *et al.*, 2018), attention and executive functions should not be disregarded in early assessments.

Our approach shares conceptual similarities with frameworks such as the Developmental-Score (D-Score) and the Global Scales of Early Development project led by the World Health Organisation ( McCray *et al.*, 2023; Weber *et al.*, 2019), which also aim to model an underlying domain-general developmental ability in early childhood. However, important differences in methodology and research focus should be noted. These global initiatives use item response theory applied to performance-based measures harmonised across multiple cohorts to produce calibrated, cross-population metrics. In contrast, our study uses PCA of parent-report measures in a single cohort to capture shared variance across three domains. Our approach is exploratory and sample-specific, focusing on the correlational structure of parent-reported outcomes rather than generating a standardised developmental metric. While the children in our sample did not complete the performance-based D-score items, limiting linkage to the D-score, van Buuren et al. (2025) recently extended the D-score framework to include both performance-based and caregiver-reported measures. Although our parent-report instruments differ from those in their analysis, their findings demonstrate that parent-report measures can contribute alongside administered tests to modelling latent developmental ability and highlight the potential for integrating parent-report and standardised assessments in future studies.

***Strengths and limitations.*** The study is unique in that we report and establish relationships amongst a range of parent-reported neuropsychological outcomes in the early years of preterm children and a term-born comparison group. In this way, we present a more comprehensive profiling of children’s abilities after preterm birth and avoid deriving conclusions about difficulties (or the lack thereof ) in only certain domains. By reporting outcomes from subscales in addition to composite scores, we also describe parent-perceived abilities with more granularity.

The study has some limitations. First, the principal component analysis may be limited by sample size inherent to the design requirement to include participants with complete datasets across measures. However, there were no statistically significant differences between those included in the principal component analysis versus the whole study population for gestational age, sex, birthweight Z-score, socioeconomic status and preterm co-morbidities. Second, although the study population is representative of that from which it was drawn, the generalisability of results for children in other settings (for example different socioeconomic circumstances) is uncertain. Third, it is possible that differences in executive functions and/or attention by parent report at this age may be detectable in a larger sample. Finally, data regarding receipt of early interventions were not systematically collected in this observational study; therefore, we do not address the potential impact of interventions on the outcomes reported here.

We propose that certain absences of group-wise differences in parental ratings may be related to performance-based measures having higher cognitive demands, potentially teasing out preterm children’s reduced abilities to meet task requirements. Alternatively, it may be that underlying differences become readily observable later in childhood. Future work is needed to assess complementarity between examiner-administered tasks and parent/caregiver ratings of outcomes, longitudinally. Parental ratings may be further biased by their family context, including presence or absence of siblings as a frame of reference, or shared response bias such as perceiving child’s abilities as uniformly positive or negative. However, certain measures such as BRIEF-P preclude shared response bias by including validity scales of negativity and consistency of ratings. Parental ratings may also depend on literacy, though we somewhat account for this in the representation of maternal education in our cohort. Nonetheless, other factors moderating cross-parental consistency in ratings should thus be investigated.

Finally, there were no term/preterm group differences on most measures, so we included all children in our correlational analyses and PCA to maximise power. However, recent work suggests that cognitive abilities ascertained using performance-based tests are more strongly interrelated in very preterm compared to term-born 5.5-year-olds ( Rapuc *et al.*, 2024); therefore if our approach is extended to performance-based measures in toddlers, it may be interesting to compare correlational structures of measures stratified by GA.

## Conclusions

At 2 years, language abilities, but not attention and executive functions, are lower in preterm children compared to term-born controls, according to parental report. The correlational structure of outcomes shows that domains are positively correlated, indicating that there is an underlying factor derivable from parental report of language, attention and executive function abilities. Our findings suggest that parental ratings can capture both domain-specific differences and broader cognitive variation, providing insight into parents’ ability to discriminate outcomes across domains and sensitively detect preterm birth-associated differences in early language development. Future work using domain-complementary standardised assessments is required to contextualise these findings.

### Ethics and consent

Ethical approval was obtained from the National Research Ethics Service, South East Scotland Research Ethics Committee (16/SS/0154, dated 5/10/2016). Informed written consent was obtained from a person with parental responsibility for each participant. Consent to participate in the study was sought from each participant only after a full explanation had been given, an information leaflet offered and time allowed for consideration. The information sheet explicitly stated “We will ask to collect information about you and your pregnancy from your medical records”. Signed participant consent was obtained by a member of the research team with training in GCP and procedures for research involving children and young people. Consent to participate was separated into two forms, one for the mother to consent to participate and one for a parent/guardian to consent for the baby/child to participate. The consent item for access to maternal medical records read “I understand that relevant sections of my medical notes and data collected during the study may be looked at by responsible individuals from the University of Edinburgh and NHS Lothian or from regulatory authorities where it is relevant to my taking part in this research. I give permission for these individuals to have access to my records”. The consent item for child medical records read “I understand that relevant sections of my child’s medical notes and data collected during the study may be looked at by responsible individuals from the University of Edinburgh and NHS Lothian or from regulatory authorities where it is relevant to my taking part in this research. I give permission for these individuals to have access to my child’s records”. The consent item for questionnaire data read “The researchers can contact me to complete questionnaires and/or interview me.” The right of the participant to refuse to participate without giving reasons was respected. All participants were free to withdraw at any time without giving reasons and without prejudicing further treatment. The study was conducted according to the principles of the Declaration of Helsinki.

## Data Availability

Data reported in this study are not publicly available due to them containing information that could compromise participant consent, which stipulates that the use of anonymised data is for studies of perinatal health that have been approved by regulatory bodies (National Research Ethics Service, South East Scotland Research Ethics Committee (16/SS/0154, dated 5/10/2016)). The terms of access to anonymised data are described in the Theirworld Edinburgh Birth Cohort Data Access and Collaboration policy available at https://reproductive-health.ed.ac.uk/theirworld-edinburgh-birth-cohort-tebc/for-researchers/data-access-and-collaboration . In brief, requests to access data will be reviewed by a study management group that will grant access based on assessment of the scientific justification of the proposed research in perinatal health and evidence of resource and capacity to analyse the data. Applications should be made via the study website above. Participant demographic, clinical, and assessment data and metadata used in this work are deposited in Edinburgh DataVault via Theirworld Edinburgh Birth Cohort (TEBC) and PREterm birth as a determinant of Neurodevelopment and COGnition in children: mechanisms and causal evidence (PRENCOG) ( https://doi.org/10.7488/e65499db-2263-4d3c-9335-55ae6d49af2b) ( Boardman & Hall, 2022). The senior author of this manuscript (JPB) is the Creator and Owner of this Edinburgh DataVault repository, where all TEBC and PRENCOG data underlying publications are deposited. Open Science Framework (OSF): Parental report of language, attention and executive functions at two years: correlational structure of measures and applications to prematurity, (DOI 10.17605/OSF.IO/XD7HR) https://www.doi.org/10.17605/OSF.IO/XD7HR ( Smikle, 2025). This OSF project contains the following extended data:
• Parent-reported cognition – STROBE Checklist. pdf • Participant Information Sheet for controls: TEBC-PIScontrols_version 2.4_20241015.pdf • Participant Information Sheet for preterms: TEBC-PISpreterms_version 2.2_20241015.pdf • Consent Form: TEBC-Maternal consent_post hospital discharge to 5 years_version 1.1_20170918 • Consent Form: TEBC-Parent or guardian consent for infant_post hosp discharge to 5 yrs_version 1.4_20201113 License: CC-By Attribution 4.0 International.

## Software availability

All analysis was conducted using open access software: R (version 4.2.3) ( https://www.r-project.org/).

Source analytic code available from: https://git.ecdf.ed.ac.uk/jbrl/parent-report-of-2-year-cognition. License: Apache 2.0.

Archived code available from DOI: 10.5281/zenodo.18711421.

License: Apache 2.0.
