# Supplementary material for: Parental report of language, attention and executive functions at two years: correlational structure of measures and applications to prematurity
Source: Wellcome Open Res. 2026 Mar 13;10:317. Originally published 2025 Jun 10. [Version 2] doi: 10.12688/wellcomeopenres.24065.2 (PMC13032106; doi:10.12688/wellcomeopenres.24065.2)
Supplement: Peer review history

# Reviewer report: Reviewer response for version 2

**DOI:** 10.21956/wellcomeopenres.28708.r151371

**Published:** 2026-04-07

**Authors:** Samson Nivins

**Affiliations:** 1 Karolinska Institutet, Stockholm, Sweden

Thank you for inviting me to review this paper titled ‘Parental report of language, attention and executive functions at two years: correlational structure of measures and applications to prematurity’ by Smikle et al., where the authors investigate the association between GA and parent reports of behavioural outcomes, and looked at their correlational structure. Authors found that GA was positively associated with language outcomes but not attention or EF; and have also reported the correlation between measures.

 The paper is very well written and clear; I command authors for that.

 I have some suggestion for improving.

 Here are some comments:

 Page 4: In the first para, I agree with authors, however, it would be great if they strengthen the argument by adding studies from Nordic countries that have showed the independent effect of preterm birth on cognitive outcomes, after accounting of genetics. In addition, you need to also reflect about the persistence of impairment across four decades based on latest JAMA Peds Meta-analysis.

 In the first para, instead of briefly stating about the rates of impairment, if you could add % alongside effect sizes would be helpful for the readers.

 In the second para, do incorporate and relate to brain development and talk about how sensory and other networks do mature. Although I agree with the text provided.

 Study Sample:

 Can you be more explicit how many children had all data available from parents, and how many did not have.

 In Table 1.

 For term born I can see the range from 36 to 42. Did you include any children from 36 weeks? Can you check.

 Although authors have selected confounders based on previous literature – I could see that substance use for e.g., tobacco use and alcohol use differ between the groups. I would strongly suggest doing an additional sensitivity test.

 Same way for e.g., GDM 2.1 vs 4.6% p value is 0.14 but pre-eclampsia 19.8 vs 5.7 why the p values is 0.43? Can you double check.

 Can you also add details of SGA, LGA, vs. AGA.

 Just for your thoughts – since you look at cognition as one of the outcomes. Do you have details of parental language spoken at home, which is known factors related to language development. Whether all family recruited are native English speakers or? Can you add those details alongside Ethnicity distribution in Table 1.

 As I have been reading your results, I was thinking whether do you have power to do knot (spline regression) analysis and can be computed easily in R programme.

 In Table 4: Why authors carried out both unrotated and rotated components. Can you add justification.

 Discussion:

 For male sex and poor language. Can you comment about different window of development between sexes.

 Its very interesting that you are not seeing difference in both attention and EF. May be too early to capture, this might be evident as they grow older. Can you talk about the maturity of frontal cortex and map to the development of EF with it.

 Can you add the reference of this line “Furthermore, de Kievet et al. (2012) showed

 that working memory mediates the relationship between birth GA and inattentive behaviours at school age”.  

 In the limitation – you should also spell out that children born at 37 and 38 weeks also tend to exhibit neurodevelopmental problems. Sorry for self-citing. If you have power you can limit the analysis to 39 GA onwards.

 (refer 1)

Is the work clearly and accurately presented and does it cite the current literature?

Yes

If applicable, is the statistical analysis and its interpretation appropriate?

Yes

Are all the source data underlying the results available to ensure full reproducibility?

Yes

Is the study design appropriate and is the work technically sound?

Yes

Are the conclusions drawn adequately supported by the results?

Yes

Are sufficient details of methods and analysis provided to allow replication by others?

Yes

Reviewer Expertise:

Pediatrics, Cognition, Preterm birth

I confirm that I have read this submission and believe that I have an appropriate level of expertise to confirm that it is of an acceptable scientific standard, however I have significant reservations, as outlined above.

## References

[1] https://bmjpublichealth.bmj.com/content/4/1/e003708.

# Reviewer report: Reviewer response for version 2

**DOI:** 10.21956/wellcomeopenres.28708.r150735

**Published:** 2026-03-27

**Authors:** Erin Andres

**Affiliations:** 1 University of Missouri, Columbia, USA

I have no further comments to make. I appreciate the authors response to the previous reviews. I especially appreciate their clear response about my question about intervention, in providing a source about the standard of care in follow-up of preterm infants and how that adds to the importance of presenting these findings from the 2 year time point. The authors very nicely argue for the analysis and focus on scalable parent report measures.

Is the work clearly and accurately presented and does it cite the current literature?

Yes

If applicable, is the statistical analysis and its interpretation appropriate?

Partly

Are all the source data underlying the results available to ensure full reproducibility?

Yes

Is the study design appropriate and is the work technically sound?

Yes

Are the conclusions drawn adequately supported by the results?

Partly

Are sufficient details of methods and analysis provided to allow replication by others?

Yes

Reviewer Expertise:

Prospective longitudinal birth cohort data for monitoring of Autism Spectrum Disorder, language acquisition, human genetic investigation of language-related phenotypes

I confirm that I have read this submission and believe that I have an appropriate level of expertise to confirm that it is of an acceptable scientific standard.

# Reviewer report: Reviewer response for version 2

**DOI:** 10.21956/wellcomeopenres.28708.r150736

**Published:** 2026-03-17

**Authors:** Dorien Emmers

**Affiliations:** 1 Katholieke Universiteit Leuven (KU Leuven), Leuven, Belgium

The authors have carefully addressed each of the comments raised. I believe this version of the manuscript is suitable for indexing.

Is the work clearly and accurately presented and does it cite the current literature?

Partly

If applicable, is the statistical analysis and its interpretation appropriate?

Partly

Are all the source data underlying the results available to ensure full reproducibility?

No

Is the study design appropriate and is the work technically sound?

Yes

Are the conclusions drawn adequately supported by the results?

Yes

Are sufficient details of methods and analysis provided to allow replication by others?

Yes

Reviewer Expertise:

early child development, field research, measurement of child development outcomes

I confirm that I have read this submission and believe that I have an appropriate level of expertise to confirm that it is of an acceptable scientific standard.

# Reviewer report: Reviewer response for version 1

**DOI:** 10.21956/wellcomeopenres.26551.r136195

**Published:** 2025-11-04

**Authors:** Erin Andres

**Affiliations:** 1 University of Missouri, Columbia, USA

Summary

 Smikle and colleagues present parent report data from a prospective longitudinal study (though data mostly from one time point is used), in which they compare 2-year outcomes related to language, executive function, and attention in toddlers who were born preterm (96) versus those born full-term (87). Their findings indicated that preterm birth predicted language outcomes on two language related parent report measures at age 2 but did not predict parent report of executive function or attention at age 2. One of their intentions in completing this analysis was to add to the literature about cognitive abilities in preterm infants using the scalable and cost-effective method of parent report. Specifically, their analyses are intended to determine if the parent report measures of the three concepts have one underlying factor to represent general cognitive function in toddlers.

 As the authors mention parent report data has been used more frequently in recent years and it is an important tool for understanding development in children with different histories. Therefore, the data and analyses presented are an important addition to the field and our overall understanding of these concepts in toddlers.

 Comments:

1. Do the authors have an additional information about if these children received any early interventions related to language? If anything can be provided for that context it would be useful for putting the overall findings into context. Given that the authors mention that these types of findings will contribute to identifying children who may need and would benefit from intervention, and that preterm infants often are monitored differently in general, it may be likely that some of the toddlers in either group may have already received some intervention.  
2. Given that the language outcomes showed the group differences and the other domains did not, the fact that two of the parent report measures related to language (CDI Word Production and VBAS receptive communication, it seems another explanation for the observed general factor (as argued by the authors) is that the measures that make up the general factor were responded to similarly by all parents. While VBAS expressive communication does meet the criteria to be part of PC1, it is the weakest relationship observed, and this can likely be explained by the authors’ findings for the adjusted models (controlling for additional demographic characteristics). The authors should include some discussion of the overlap in these findings in their limitations discussion.
3. An addition of further analyses concerning the non-significant adjusted model for the VBAS expressive communication could lead to an interesting discussion about which of the additional variables accounted for more variance (age at time of testing, infant sex, or maternal education).
4. The authors conclusions that their work “provides insight into parent’s approach to discriminate outcomes across domains” should be expanded on prior to making this conclusion. The presentation of results as is do not make a good argument that the data provide this insight. If this conclusion is meant to support the finding concerning group differences for language but not executive function or attention, this should be more clear in the conclusion.

Is the work clearly and accurately presented and does it cite the current literature?

Yes

If applicable, is the statistical analysis and its interpretation appropriate?

Partly

Are all the source data underlying the results available to ensure full reproducibility?

Yes

Is the study design appropriate and is the work technically sound?

Yes

Are the conclusions drawn adequately supported by the results?

Partly

Are sufficient details of methods and analysis provided to allow replication by others?

Yes

Reviewer Expertise:

Prospective longitudinal birth cohort data for monitoring of Autism Spectrum Disorder, language acquisition, human genetic investigation of language-related phenotypes

I confirm that I have read this submission and believe that I have an appropriate level of expertise to confirm that it is of an acceptable scientific standard, however I have significant reservations, as outlined above.

# Response

**Published:** 2026-02-20

**Authors:** Rebekah Smikle

Thank you for the opportunity to respond to reviewer comments. We appreciate their feedback and suggestions for improvement. Below we provide a point-by-point response to each comment. Reviewer comments are reproduced verbatim, followed by our responses in italics.

1. Do the authors have an additional information about if these children received any early interventions related to language? If anything can be provided for that context it would be useful for putting the overall findings into context. Given that the authors mention that these types of findings will contribute to identifying children who may need and would benefit from intervention, and that preterm infants often are monitored differently in general, it may be likely that some of the toddlers in either group may have already received some intervention. 

*All of the children in the study were eligible for assessment of speech, language and communication according to the universal Child Health Surveillance Programme Pre-School in Scotland. In addition, the preterm group is offered follow-up to 2-years by the National Health Service. It is possible that either pathway could have triggered referral to speech and language services. We do not have data about interventions offered to either group between hospital discharge or two years. We agree that assessing the efficacy of interventions using parent report would be interesting future work, but it was beyond the scope of this work.* *We have now added this point to the Limitations section: “Finally, data regarding receipt of early interventions were not systematically collected in this observational study; therefore, we do not address the potential impact of interventions on the outcomes reported here.”* *Importantly, because this was an observational study designed to examine associations between gestational age at birth and 2-year neuropsychological outcomes, the primary conclusions regarding group differences remain unchanged. *  

1. Given that the language outcomes showed the group differences and the other domains did not, the fact that two of the parent report measures related to language (CDI Word Production and VBAS receptive communication, it seems another explanation for the observed general factor (as argued by the authors) is that the measures that make up the general factor were responded to similarly by all parents. While VBAS expressive communication does meet the criteria to be part of PC1, it is the weakest relationship observed, and this can likely be explained by the authors’ findings for the adjusted models (controlling for additional demographic characteristics). The authors should include some discussion of the overlap in these findings in their limitations discussion.

*We thank the reviewer for this insight. We have amended the Discussion accordingly:* *“Although birth GA was associated with language outcomes, language measures contributed relatively weakly to the general factor. Specifically, VABS expressive communication, while meeting criteria for inclusion in PC1, showed the lowest loading and was also the only language measure for which association with birth GA was attenuated after adjusting for covariates. This overlap suggests that while some variance in this measure is accounted for by the domain-general factor, other variance may reflect demographic influences, and that GA-related language differences are unlikely to be the primary drivers of the domain-general factor.* *”*  

1. An addition of further analyses concerning the non-significant adjusted model for the VBAS expressive communication could lead to an interesting discussion about which of the additional variables accounted for more variance (age at time of testing, infant sex, or maternal education).

*We thank the reviewer for this suggestion. We have included this additional analysis in the Results section titled ‘Associations between gestational age at birth and neuropsychological outcome’ after the initial analysis of VABS expressive communication and GA in adjusted models:* *“In models adjusted for sex, age at testing, and maternal education, a similar pattern of results was observed, except that the association between GA and VABS expressive communication was no longer statistically significant (β = 0.130, SE 0.078, p FDR = 0.100). Post-hoc analyses revealed that this attenuation was driven by sex (β = -0.526, SE 0.078, p = 0.008), which accounted for an additional 9.9% of variance in VABS expressive communication, whereas maternal education and age at testing did not significantly contribute.”* *In the Discussion section, after discussing our results about language and GA, we have added the following: “The association between birth GA and expressive language was not statistically significant when adjusting for sex, consistent with previous research showing that male sex is strongly predictive of poorer language and general cognitive outcomes in children at 2 years of age* *(Haider et al., 2025; Linsell et al., 2015; O’Driscoll et al., 2018)* *.”*    

1. The authors conclusions that their work “provides insight into parent’s approach to discriminate outcomes across domains” should be expanded on prior to making this conclusion. The presentation of results as is do not make a good argument that the data provide this insight. If this conclusion is meant to support the finding concerning group differences for language but not executive function or attention, this should be more clear in the conclusion.

*Thank you for this suggestion on clarity. We have revised this line of the conclusion to state: “Our findings suggest that parental ratings can capture both domain-specific differences and broader cognitive variation, providing insight into parents’ ability to discriminate outcomes across domains and sensitively detect preterm birth-associated differences in early language development. Future work using domain-complementary standardised assessments is required to contextualise these findings.”*

# Reviewer report: Reviewer response for version 1

**DOI:** 10.21956/wellcomeopenres.26551.r131736

**Published:** 2025-09-08

**Authors:** Dorien Emmers

**Affiliations:** 1 Katholieke Universiteit Leuven (KU Leuven), Leuven, Belgium

**Summary:**

 This paper examines cognitive outcomes in toddlers born preterm, who are at heightened risk of impairments in general ability and specific domains such as language, attention, and executive function. Their scores are compared to term-born peers. While standardized observational assessments such as the Bayley Scales of Infant and Toddler Development (fourth edition) are commonly used, parent-report tools are gaining traction due to their ecological validity, scalability, and cost-effectiveness, though little is known about their effectiveness in capturing language, attention, and executive function deficits at age of two. The current study investigates whether parent-reported measures of language, attention, and executive function are correlated with gestational age at birth and explores whether a latent cognitive development factor underlies the various measures.

**Comments:**

1. I believe the authors could strengthen their discussion of the relevance of their work. For example, when they note that “parent-report measures have gained traction… because of their high ecological value, relevance to adaptive behaviour in everyday settings (Loe et al., 2015), and because of cost-effectiveness and scalability,” this point is central to justifying the study’s contribution. Expanding on this explanation and supporting it with a broader range of references would provide a more compelling rationale for the use of parent-report measures in this context.
2. Given that the authors are investigating whether a latent factor underlies the different parent-report measures, it would be important to situate their work in relation to existing approaches such as the D-score and the Global Scales of Early Development (GSED) for children under age three. How does their approach compare to these frameworks, and what are the respective advantages and limitations? Additionally, the authors might consider linking their data to the openly accessible D-score dataset, which could both strengthen the study’s external validity and enhance its contribution to the broader field.
3. It would be helpful to expand the discussion of the extent to which the developmental domains of effortful control (attention) and executive functioning overlap or remain distinct, supported by references to relevant literature.
4. Regarding the study sample selection, it remains unclear whether the authors worked with the full eligible population or a random sample. The manuscript would benefit from a clearer and more detailed description of the sampling strategy.
5. With respect to the data analysis, several extensions could add value. First, given that the dataset is longitudinal, it would be informative to present how developmental gaps evolve over age and how the measures correlate with earlier developmental outcomes. Second, after constructing developmental factors, the authors do not use these further in their analyses. It would strengthen the paper to show how factor scores differ between term- and preterm-born children, and whether they are associated with demographic or socioeconomic characteristics.
6. Finally, the interpretation of the 3-factor solution is not fully convincing. It appears to largely reflect the specific measures included rather than a deeper latent structure. The authors should expand their discussion to clarify the relevance of these factor results and what they contribute to our understanding of developmental outcomes in preterm children.

Is the work clearly and accurately presented and does it cite the current literature?

Partly

If applicable, is the statistical analysis and its interpretation appropriate?

Partly

Are all the source data underlying the results available to ensure full reproducibility?

No

Is the study design appropriate and is the work technically sound?

Yes

Are the conclusions drawn adequately supported by the results?

Yes

Are sufficient details of methods and analysis provided to allow replication by others?

Yes

Reviewer Expertise:

early child development, field research, measurement of child development outcomes

I confirm that I have read this submission and believe that I have an appropriate level of expertise to confirm that it is of an acceptable scientific standard, however I have significant reservations, as outlined above.

## References

[1] : The D-score: a metric for interpreting the early development of infants and toddlers across global settings. *BMJ Global Health* .2019;4(6) : 10.1136/bmjgh-2019-001724 10.1136/bmjgh-2019-001724 PMC688255331803508

[2] : Protocol for validation of the Global Scales for Early Development (GSED) for children under 3 years of age in seven countries. *BMJ Open* .2023;13(1) : 10.1136/bmjopen-2022-062562 10.1136/bmjopen-2022-062562 PMC988487836693690

[3] : Executive function and effortful control—Similar and different evidence from big data analysis. *Frontiers in Psychology* .2022;13: 10.3389/fpsyg.2022.1004403 10.3389/fpsyg.2022.1004403 PMC979486636591081

# Response

**Published:** 2026-02-20

**Authors:** Rebekah Smikle

Thank you for the opportunity to respond to reviewer comments. We appreciate their feedback and suggestions for improvement. Below we provide a point-by-point response to each comment. Reviewer comments are reproduced verbatim, followed by our responses in italics. Comments:

1. I believe the authors could strengthen their discussion of the relevance of their work. For example, when they note that “parent-report measures have gained traction… because of their high ecological value, relevance to adaptive behaviour in everyday settings (Loe et al., 2015), and because of cost-effectiveness and scalability,” this point is central to justifying the study’s contribution. Expanding on this explanation and supporting it with a broader range of references would provide a more compelling rationale for the use of parent-report measures in this context.

*We thank the reviewer for this suggestion on emphasising the value of this study. We have revised the relevant paragraph of the Introduction also including supporting references:* *“Characterising cognition in the first few years of life is important for detecting developmental difficulties and for stratifying preterm infants at risk who may benefit from targeted early interventions. In several countries, neurodevelopmental assessment at 2 years of age is part of standard follow-up care for preterm infants, as this timepoint represents an important milestone for identifying major neurological and sensory disabilities* *(Marlow, 2004; Seppänen et al., 2024)* *. Around 2 years of age, there is major growth in cognition and language, and this acquisition marks the transition from infancy to early childhood. Cognitive ability at 2 years is typically assessed in clinical and research settings using standardised examiner-administered developmental assessments, such as the Bayley Scales of Infant and Toddler Development-Fourth Edition (Bayley-4). In recent years, parent-report measures have gained traction to assess outcomes in both settings because of their high ecological value, relevance to adaptive behaviour in everyday settings, and because of cost-effectiveness and scalability* *(Baker et al., 2013; Johnson et al., 2008, 2019; Loe et al., 2015)* *.* *Unlike examiner-administered assessments, parent-report measures capture children’s abilities across multiple environments and everyday activities, reflecting functional skills that may not be apparent in structured testing. Parental questionnaires also mitigate the practical constraints of data collection, particularly for children with the most pronounced impairments who may be unable to fully engage with standardised testing protocols. For example, the Parent Report of Children’s Abilities-Revised (PARCA-R) provides a validated assessment of children’s cognitive and language development at 24–27 months (* *Johnson et al., 2019* *). It is useful as an endpoint in randomised controlled trials of interventions designed to improve outcomes after preterm birth (* *Dargaville et al., 2023* *;* *Moore et al., 2023* *). For a more comprehensive view of a child’s functioning and to identify subtle and specific deficits, adopting a transdiagnostic neuropsychological approach assessing cognition across multiple domains is recommended* *(Anderson, 2014; Astle et al., 2022)* *. Previous research has shown that parental report at 2 years can be used to identify transdomain clusters of neurodevelopment at scale, replicates across countries, has clinical validity and is useful for investigating upstream determinants of neurodevelopmental outcomes* *(Haider et al., 2025)* *. Evidently, parent-report questionnaires with adequate psychometric properties may serve as scalable, complementary tools to labour- and time-intensive direct testing* *, but less is known about whether these measures of other domains relevant to preterm birth, including attention and executive function, capture differences in toddlerhood. Yet, to better understand the nature of impairments in children born preterm and avoid underestimating difficulties experienced in the early years, it is necessary to use instruments that measure performance in multiple domains.”*  

1. Given that the authors are investigating whether a latent factor underlies the different parent-report measures, it would be important to situate their work in relation to existing approaches such as the D-score and the Global Scales of Early Development (GSED) for children under age three. How does their approach compare to these frameworks, and what are the respective advantages and limitations? Additionally, the authors might consider linking their data to the openly accessible D-score dataset, which could both strengthen the study’s external validity and enhance its contribution to the broader field.

*We appreciate the reviewer’s suggestion for broader contextualisation of our work. We have added the following text to the Discussion:* *“Our approach shares conceptual similarities with frameworks such as the Developmental-Score (D-Score) and the Global Scales of Early Development project led by the World Health Organisation* *(McCray et al., 2023; Weber et al., 2019)* *, which also aim to model an underlying domain-general developmental ability in early childhood. However, important differences in methodology and applications should be noted. These global initiatives use item response theory applied to performance-based measures harmonised across multiple cohorts to produce calibrated, cross-population metrics. In contrast, our study uses PCA of parent-report measures in a single cohort to capture shared variance across three domains. Our approach is exploratory and cohort-specific, focusing on the correlational structure of parent-reported outcomes rather than generating a standardised developmental metric. While the children in our sample did not complete the performance-based D-score items, limiting linkage to the D-score, van Buuren et al.* *(2025)* *recently extended the D-score framework to include both performance-based and caregiver-reported measures. Although our parent-report instruments differ from those in their analysis, these findings further demonstrate that parent-report measures can contribute alongside administered tests to modelling latent developmental ability and highlight the potential for integrating parent-report and standardised assessments in future studies.”* *While both the D-score framework and our approach seek to capture domain-general ability in some regard, they differ substantially in methodology, data source and intended application. For this reason, we did not consider a direct comparison of advantages and limitations between these approaches as appropriate. Similarly, the measures included in our dataset do not map directly onto the items used to generate D-scores, and harmonisation would require assumption about item equivalence and scaling that are beyond the scope of this observational study. Nonetheless, our work provides complementary information, demonstrating that a domain-general factor can be derived from parent-report measures. *  

1. It would be helpful to expand the discussion of the extent to which the developmental domains of effortful control (attention) and executive functioning overlap or remain distinct, supported by references to relevant literature.

*We thank the reviewer for this suggestion. In the Discussion, after contextualising our findings on the associations between gestational age at birth and these domains, we have added this paragraph:* *“As higher-order processes required for goal-directed behaviour, attention and executive functions are separable but interrelated constructs. The development of attentional skills overlaps with that of executive functioning in the early years, with a core literature arguing that the maturation of attentional capacity may contribute to various emerging executive functions* *(Garon et al., 2008; Reynolds & Romano, 2016)* *. In the present study, significant cross-domain correlations were observed, particularly between ECBQ attentional measures and BRIEF-P Working memory and Inhibit, consistent with this developmental interdependence. Previous research in both preterm and typically developing children shows that some individuals with low working memory performance are frequently rated by teachers and parents as having elevated attention problems* *(Aarnoudse-Moens et al., 2013; Gathercole et al., 2008)* *. Furthermore, de Kievet et al.* *(2012)* *showed that working memory mediates the relationship between birth GA and inattentive behaviours at school age. At the same time, the rotated PCA solution identified distinct components corresponding to each domain, in line with established two-factor models of attentional control and executive function that sample multiple subdomains of these processes in early childhood* *(Deodhar & Bertenthal, 2023)* *. Together, these findings suggest that while attention and executive functions are closely related, they retain measurable differentiation at 2 years of age.”*
 

1. Regarding the study sample selection, it remains unclear whether the authors worked with the full eligible population or a random sample. The manuscript would benefit from a clearer and more detailed description of the sampling strategy.

*We thank the reviewer for this note on clarity regarding the sampling strategy. In the Results section titled ‘Participant characteristics’, we noted that “The study sample consisted of 183 mother and child dyads (96 preterm and 87 term-born children)”. In the Methods subsection titled ‘Study sample’, we described recruitment and exclusion criteria for the Theirworld Edinburgh Birth Cohort (TEBC), from which the current sample was drawn. The current study sample included all children from the TEBC who had at least one parent-report measure at 2 years of age. To clarify the derivation of the analysed sample, we have added: “Infants were invited for assessment at 2 years of age. We included all children who attended 2-year follow-up and had at least one parent-report measure available in any of the domains of language, attention or executive functions (N = 183).”*  

1. With respect to the data analysis, several extensions could add value. First, given that the dataset is longitudinal, it would be informative to present how developmental gaps evolve over age and how the measures correlate with earlier developmental outcomes. Second, after constructing developmental factors, the authors do not use these further in their analyses. It would strengthen the paper to show how factor scores differ between term- and preterm-born children, and whether they are associated with demographic or socioeconomic characteristics.

*We agree that longitudinal analyses examining developmental trajectories and associations with earlier outcomes would be of interest and is planned as the children reach school age (5-year and 7-year data collection is underway). However, the present study was designed to specifically describe parent-reported neuropsychological outcomes at 2 years of age. We have added a further justification for the utility of assessment at the 2-year timepoint to the Introductory paragraphs: “Characterising cognition in the first few years of life is important for detecting developmental difficulties and for stratifying preterm infants at risk who may benefit from targeted early interventions. In several countries, neurodevelopmental assessment at 2 years of age is part of standard follow-up care for preterm infants, as this timepoint represents an important milestone for identifying major neurological and sensory disabilities* *(Marlow, 2004; Seppänen et al., 2024)* *and identifying which toddlers may benefit from additional support. Around 2 years of age, there is major growth in cognition and language, and this acquisition marks the transition from infancy to early childhood.* *”* *After deriving the principal component 1 (PC1) scores, we assessed their correlations with gestational age at birth as described in the Results: “The unrotated PC1 scores were not correlated with gestational age at birth (rho = -0.12, p = 0.23)”. At the reviewer’s request, we have included further analyses of how these scores differed between groups, and whether they are associated with socioeconomic and demographic characteristics in the Results section titled ‘Correlational structure of neuropsychological outcome measures at 2 years and principal component analysis’: “The unrotated PC1 scores were not correlated with gestational age at birth ( rho = -0.12, p = 0.23) and did not differ between preterm and term-born children in groupwise analyses (t(90.9) = 0.27, p = 0.79). In a multivariable linear regression, we also observed no significant association of PC1 scores with gestational age at birth (β <0.001, SE 0.101, p = 0.998), sex (male: β = -0.147, SE 0.206, p = 0.479), maternal education (university-level or higher: β = 0.349, SE 0.253, p = 0.170) or age at testing (β = -0.085, SE 0.105, p = 0.421).”* *We offer an interpretation and implications of these findings in the Discussion:* *“Notably, the domain-general factor was not associated with birth GA, infant sex or maternal education, suggesting that the effects of prematurity and demographic influences are not pervasive across parent-reported cognitive domains at 2 years.”*
 

1. Finally, the interpretation of the 3-factor solution is not fully convincing. It appears to largely reflect the specific measures included rather than a deeper latent structure. The authors should expand their discussion to clarify the relevance of these factor results and what they contribute to our understanding of developmental outcomes in preterm children.

*We thank the reviewer for highlighting the need to clarify the interpretation and relevance of the 3-factor solution. In the Results, we previously acknowledged that the rotated principal components broadly aligned with the measures included: “Extracting three rotated components yielded a loading pattern mainly consistent with the measurement instruments: PC1 showed preferential loadings from BRIEF-P scores (executive functions), PC2 VABS and CDI (language); PC3 on the two ECBQ attentional scores.”* *We have incorporated the point into the revised the Discussion as follows:* *“Examination of the rotated PCs clarifies domain-specific contributions. One component primarily reflected executive functions (all measured via BRIEF-P), another language (measured across VABS and CDI) and a third attention (both ECBQ subscales). For the language factor, the alignment of measures from different instruments suggests that this component captures a coherent developmental domain rather than being an artefact of a single questionnaire. That unique measures of a single domain (specifically VABS and CDI for language) showed similarly strong loadings for a specific rotated PC indicates that similar constructs are “mapping” to specific components, beyond those from a single assessment tool. Although the executive function and attention components were derived from subscales within single instruments, the pattern of loadings was consistent with theoretically defined domains* *(Deodhar & Bertenthal, 2023)* *, supporting the interpretation that the solution reflects meaningful developmental constructs rather than purely instrument-specific variance.* *The uneven distribution of loadings across domains may relate to strong intercorrelations within particular domains in this age group or in relation to prematurity, as well as more available measures for particular domains (e.g. BRIEF-P for executive functions) than others (e.g. attention).* *Beyond reflecting the instruments included, the 3-component solution has developmental implications. By 2 years of age, language, attention and executive functions appear measurably differentiated in parent-report data, while still sharing substantial variance suggestive of general cognitive processes. Together, these findings suggest that while parent report reflects consistent individual differences across domains, it does not capture the impact of prematurity in driving differences in domains beyond language. Nonetheless, as children who show difficulties in one domain are more likely to experience difficulties in others and generalised cognitive difficulties on direct measures are well-reported in the literature* *(Anderson, 2014; Brydges et al., 2018; Mulder et al., 2009; Twilhaar et al., 2018)* *, attention and executive functions should not be disregarded in early assessments.* *”* *Finally, in the conclusion, we now state: “Our findings suggest that parental ratings can capture both domain-specific differences and broader cognitive variation, providing insight into parents’ ability to discriminate outcomes across domains and sensitively detect preterm birth-associated differences in early language development. Future work using domain-complementary standardised assessments is required to contextualise these findings.”*
